# Local and global analysis of macromolecular atomic displacement parameters

**DOI:** 10.1107/S2059798320011043

**Published:** 2020-09-22

**Authors:** Rafiga C. Masmaliyeva, Kave H. Babai, Garib N. Murshudov

**Affiliations:** a Institute of Molecular Biology and Biotechnologies ANAS, Baku, Azerbaijan; b R.I.S.K. Scientific Production Company, Baku, Azerbaijan; c Structural Studies, MRC Laboratory of Molecular Biology, Francis Crick Avenue, Cambridge CB2 0QH, United Kingdom

**Keywords:** refinement, validation, atomic displacement parameters, inverse-gamma mixture model, Bayesian statistics, *ToBvalid*

## Abstract

This paper describes the global and local analysis of atomic displacement parameters (ADPs) of macromolecules in X-ray crystallography. The distribution of ADPs is shown to follow the shifted inverse-gamma distribution or a mixture of these distributions. The mixture parameters are estimated using the expectation–maximization algorithm. In addition, a method for the resolution- and individual ADP-dependent local analysis of neighbouring atoms has been designed. This method facilitates the detection of mismodelled atoms, heavy-metal atoms and disordered and/or incorrectly modelled ligands. Both global and local analyses can be used to detect errors in atomic models, thus helping in the (re)building, refinement and validation of macromolecular structures. This method can also serve as an additional validation tool during PDB deposition.

## Abstract 2

Macromolecular atomic *B*-value distributions have been modelled using a mixture of shifted inverse-gamma distributions. *B*-value and resolution-dependent local ADP differences have also been applied for the validation of heavy atoms and ligands.

## 1. Introduction   

The ever-increasing numbers of macromolecular structures solved by crystallographic and cryoEM methods, and deposited in the PDB (Berman *et al.*, 2000 ▸; Lawson *et al.*, 2016 ▸), require statistically robust and automatic tools for refinement (Sheldrick, 2008 ▸; Adams *et al.*, 2010 ▸; Global Phasing, 1997 ▸; Murshudov *et al.*, 2011 ▸), validation (Read *et al.*, 2011 ▸) and deposition (Adams *et al.*, 2019 ▸). In general, it is relatively intuitive, although challenging, to design tools for the valid­ation of atomic positional parameters, as they should comply with the basic structural and chemical properties of macromolecules, and there are a number of popular tools designed to do just this (Vriend, 1990 ▸; Laskowski *et al.*, 1993 ▸; Vaguine *et al.*, 1999 ▸; Joosten *et al.*, 2012 ▸; Williams *et al.*, 2018 ▸). Designing such tools for ADP validation is less intuitive and, although the importance of this problem has been stressed by many authors (Rupp, 2009 ▸; Merritt, 2011 ▸, 2012 ▸), there are currently no widely used tools to check and validate ADPs. One of the potential reasons is that ADPs reflect many shortcomings in the modelling such as crystal deficiencies (for example anisotropy, modulation and imperfection of crystals), inaccurate assumptions in data acquisition and processing, modelling problems (modelling the mobility of molecules using individual ADPs is essentially equivalent to the assumption that the atoms are oscillating independently around their central position and such oscillation is harmonic, and moreover that all unit cells behave in exactly the same way), and the intrinsic mobility of atoms within molecules and of molecules within crystals (Kuhs, 2003 ▸). Several reports have described the use of the ADP distribution as a validation criterion (Hirshfeld, 1976 ▸; Carugo & Argos, 1998 ▸; Yang *et al.*, 2016 ▸; Carugo, 2018 ▸). These papers utilize the fact that, to a certain degree, ADPs represent the uncertainty of atomic positions (Schneider *et al.*, 2014 ▸; Yang *et al.*, 2016 ▸). Using the simple fact that *B* values are proportional to the variances of the distribution of atoms around their central position and using the inverse-gamma distribution as a conjugate prior for data from a normal distribution (O’Hagan & Forster, 2004 ▸), Masmaliyeva & Murshudov (2019 ▸) proposed modelling the behaviour of ADPs using a shifted inverse-gamma distribution (SIGD). They also demonstrated that there are a number of PDB entries where the *B* values exhibit a multimodal distribution. There may be a number of reasons for such behaviour, which include the following.

(i) It is an intrinsic property of molecules within their environment (crystal or multi-domain/multi-subunit structures in cryoEM), where different components (subunits/domains) have a different number of neighbours to interact with. In such cases, different subunits/domains may have different levels of mobility, and this can be reflected in the *B*-value distribution. It can be expected that the ADPs of each structural unit will behave as an SIGD with different parameters.
(ii) Some parts of the model (loops, ligands or even domains) may have been placed incorrectly. Essentially, such behaviour indicates that there is very weak or no evidence to support the presence of these parts of the structures, and as such they should be considered with extreme care.

If it is assumed that the noise level in the map is approximately constant over the unit cell, then it can be claimed that the local signal-to-noise ratio depends on the height of the local average electron density and that this in turn depends on the local mobility of molecules. Therefore, it can be expected that (i) if atoms are placed in incorrect positions, then during refinement their *B* values will increase dramatically to reflect the absence of the density, as the signal-to-noise ratio in these regions is close or equal to zero, and (ii) if two or more domains/subunits have different intermolecular and/or crystal contacts, then they will have different ADPs reflecting their mobility, thus reducing the signal-to-noise ratio and making the interpretation of such regions very difficult. In both cases there will be multiple modes of ADP distribution, and correspondingly the signal-to-noise ratio will be different. This means that at least for some crystal structures, the local signal-to-noise ratio and therefore the local resolution will vary over the unit cell; the local resolution will have a distribution corresponding to the ADP distribution.

In this work, we model multimodal ADP distributions as a mixture of SIGDs, which can potentially be used further to identify mismodelled and/or structurally compact regions. This fact, among several other odd behaviours of ADPs, has been described by Rupp (2009 ▸) in his fine textbook on biomacromolecular crystallography.

Although the modelling of the overall ADP distribution is a good technique for the identification of suspicious/interesting regions of crystal structures, it does not allow the identification of individual mismodelled atoms, residues or ligands. To address this problem, we consider local ADP differences in a given crystal structure. In general, it is reasonable to assume that if two atoms are close to each other in space, then their mobility and ADPs should be similar. This makes sense if we consider molecules, including waters, as an elastic network (Tirion, 1996 ▸); an oscillating atom has an almost immediate effect on its surroundings. Moreover, if the atoms have been modelled correctly, then all factors influencing the ADPs of an atom should also influence the neighbouring atoms. Therefore, dramatic differences between the ADPs of atoms close to each other in 3D space may mainly be owing to different occupancies of the atoms and/or a different atom identity, *i.e.* heavy atoms may have been modelled as light atoms or vice versa.

One of the problems is that the meaning of the similarity of two ADP values is not entirely clear. For example, depending on the (local) resolution, the difference between 100 and 150 Å^2^ can be less significant than the difference between 10 and 15 Å^2^. Moreover, the resolution will also affect the significance of these differences. Therefore, to analyse the differences between *B* values of atoms, the resolution, as well as the ADPs, needs to be accounted for. Wang (2018 ▸) uses a similar idea to analyse the occupancies of atoms of different elements in crystals. Here, this idea is used to calculate the differences between ADPs as well as the potential adjustment of occupancies to make the ADPs of neighbouring atoms similar.

### 1.1. Organization of the paper   

Firstly, the mathematical formulation for modelling the ADP distribution using mixed SIGDs is described and the formulation for the analysis of local differences is then given. Finally, the described methods are applied to re-refined structures from the PDB and the results are analysed.

## 2. Materials and methods   

### 2.1. Global ADP analysis   

Multimodal ADP distributions are modelled using a mixture of SIGDs. This distribution has the form
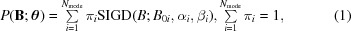
where ***B*** is a vector of observations, 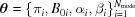 is the vector of parameters and π_*i*_ is the probability of mode *i*. *N*_mode_ is the number of modes and SIGD has the form
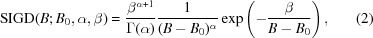
where Γ(α) is the Gamma function and *B*_0_, β and α are the shift, scale and shape parameters, respectively. The use of this function to model macromolecular ADP distributions was suggested by Dauter *et al.* (2006 ▸). It was later used by Negroni *et al.* (2010 ▸), and its utility for modelling ADP distributions was demonstrated by Masmaliyeva & Murshudov (2019 ▸).

The expectation–maximization algorithm (EM) described by Bishop (2006 ▸) is used for the estimation of the parameters of the distribution defined in (1) and (2). The direct application of the EM algorithm to the mixture of SIGDs turned out to be unstable. Therefore, the parameters were estimated in four steps.

(i) Convert the ADP distribution to a peak-height distribution (PHD).
(ii) Use the Silverman (1981 ▸) algorithm as implemented in the *SciPy* package to find the number and the centroids of the clusters.
(iii) Using the found number and the initial centroids of the modes, fit the mixture of Gaussians into the PHD.
(iv) Starting with the parameters found in the previous steps, estimate the parameters of the mixture of SIGDs using the EM algorithm (see Appendix *A* [App appa]).

In an ideal case, the minimal *B*_0_ should be close to 0. However, in practice this is rarely the case. The main reason for this seems to be that during the scaling of unmerged intensities with each other the overall *B* value is not defined and can change arbitrarily. If crystals did not change during data collection, then taking one of the images as a reference for scaling would be sufficient. However, owing to radiation damage crystals do change depending on the radiation dose, and taking any of the images as a reference will give an over/underestimation of the resultant overall *B* values. This problem can be fully resolved if unmerged intensities are used for atomic model refinement with radiation dose-dependent *B* values as parameters. It also should be mentioned that *B*_0_ as estimated using formulas (1) and (2) could be used as the safest sharpening/blurring parameter.

Accurate map sharpening/blurring requires local mobilities to be accounted for as well as the local signal-to-noise ratio. It is our view that for atomic model refinement the observed data should be used without any doctoring of the data; however, for the visually best map calculations it is necessary to weight Fourier coefficients according to the signal-to-noise ratio and sharpen/blur according to local mobility. Treatment of this problem is outside the scope of this work and will be dealt with in the future.

### 2.2. Peak heights and local ADP analysis   

For analysis of the relative occupancies of neighbouring atoms, the peak heights of point atoms with a given resolution and ADP are considered. In reality, the noise level on the amplitudes and phases as well as the weights used in the map calculations should also be accounted for. For simplification, these factors are ignored. For a Gaussian point with an ADP equal to *B*,
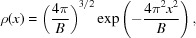
for which the scattering factor is *f*(*s*) = exp(−*B* *s*_2_/4), the peak height at the centre of the atom at a given resolution is (Chapman, 1995 ▸)
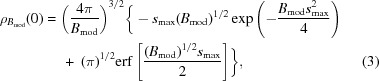
where *s*_max_ = 1/*d*_max_ is the maximum resolution, *B*_mod_ is the ADP, erf is the error function (for a survey of special functions, see Abramowitz & Stegun, 1965 ▸). Masmaliyeva & Murshudov (2019 ▸) used (3) to demonstrate that there is a resolution-dependent effect on the PHD. If two atoms with ADPs equal to *B*_1_ and *B*_2_ are considered, then the question can be posed: how much should the occupancy of the second atom be changed so that the peak height becomes the same as a fully occupied first atom? This can be expressed trivially as
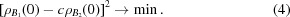
It is solved for *c* to give
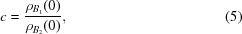
which for point atoms expanded with (3) results in
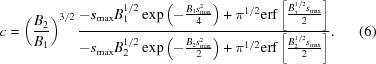
Expressions (5) and (6) can also be trivially obtained by a simple division of the expressions for peak heights for two atoms.

When *s*_max_→∞ this formula converges to
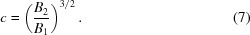
Note that the optimal occupancy value is achieved when (4) becomes zero, meaning that by changing the occupancies, the peak heights at the centre of atoms could be changed arbitrarily. Possible minimum and maximum values of the estimated relative occupancies are *c* = 0 and *c* = ∞, which are achieved when *B*_2_ = 0 and *B*_1_ = 0, respectively. Obviously, there is no physical meaning for an infinite relative occupancy; it is an artefact of using peak heights at the centre as a guide for atomic identity.

Since the atomic ADP affects the density of the atom everywhere, it might be better to use the total density differences to evaluate occupancies. We would like to find the best occupancy for the second atom so that its total density is similar to the first atom,
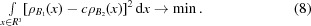
Using Parseval’s theorem (ignoring constants),
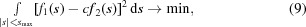
where *f*_1_(*s*) and *f*_2_(*s*) are scattering factors for the atoms. Solving (9) for *c* gives
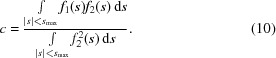
For point atoms with ADP equal to *B* this can be written as
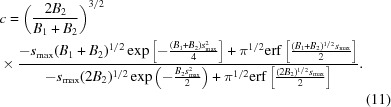
Note that when *s*_max_→∞ this formula becomes
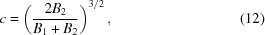
which could be used as a limiting case of occupancy estimation. Note that the maximum relative occupancy estimated using expression (11) would be achieved when *B*_1_ = 0 and *s*_max_ = ∞, which gives *c* = 2^3/2^ ≃ 2.83, meaning that in general this method will underestimate the occupancy of atoms/ligands/residues. The minimum of (11) is achieved when *B*_2_ = 0, which gives *c* = 0.

No value of *c* can make the expression in (11) equal to zero unless *B*_1_ = *B*_2_. This means that the only valid explanation of the density is using the correct atoms, which may never be possible.

Formulas (6) and/or (11) can be used for a quick check of the correctness of the elements, for example for Asn, Gln and His side-chain orientations. This will only work if the data resolution is sufficiently high and the side chains are well defined. In such cases, there will be other atoms around the side chains of these residues that make hydrogen bonds to them. Therefore, the local hydrogen-bonding network can be used to correct the orientation of Asn, Gln and His side chains (Chen *et al.*, 2010 ▸).

We would like to stress that the occupancies derived using expressions (6) and (11) are not a replacement for refined atomic occupancies, although they can be used as a starting point for occupancy refinement. These formulas are expressions for local ADP differences. It also should be noted that these formulas can be modified to account for the experimental method-dependent atomic scattering factors (see Section S1 of the supporting information). In this work and the associated software, we do not account for the scattering factors as we are interested in ‘local ADP differences’ and using point Gaussian atoms seems to be sufficient for this particular purpose.

### 2.3. Data from the PDB   

All PDB entries solved by X-ray crystallography as of November 2019, for which experimental data were available, were downloaded from the PDB and refined using *REFMAC*5 (Kovalevskiy *et al.*, 2018 ▸) as distributed within the *CCP*4 software suite (Winn *et al.*, 2011 ▸). The total number of such entries is 127 708. All structures were refined using the same software to make sure that all of the ADPs had been refined consistently using the same software (other refinement software could also be used; see, for example, Adams *et al.*, 2010 ▸; Sheldrick, 2008 ▸; Global Phasing, 1997 ▸). For further analysis, we used only the models for which the high-resolution diffraction limit is between 1.5 and 3 Å. To avoid dealing with structures refined using noncrystallographic symmetry constraints, the use of which is not always clear from the PDB, we removed virus structures. Of the remaining models, we were able to refine 90 840 automatically. Reasons for refinement failure include (i) the ligand that is present in the PDB file was not in the CCP4 monomer library (Long *et al.*, 2017 ▸) at the time of re-refinement, which was the most common case, (ii) the absence of experimental data and (iii) space-group inconsistencies between the PDB and data files. We also excluded cases with *R* factors of >0.3. Table 1 ▸ gives a short summary of the selection of PDB entries. Table 2 ▸ lists the example PDB entries used in this work. It should be stressed that the aim of this contribution is not to criticize a particular PDB entry; rather, we would like to highlight the shortcomings of the techniques used at the time of the elucidation of these structures, and the necessity of remodelling and re-refinement as new technologies become available.

It should be noted that the data from the PDB-REDO databank (Joosten *et al.*, 2012 ▸) could also be used in the analysis. In practice, if a particular PDB entry is of interest, we would recommend using, if available, the best refined atomic model from the PDB-REDO databank.

## 3. Results and discussion   

The examples below aim to demonstrate three aspects of ADPs: (i) the modelling of multimodal distributions, (ii) the identification of mismodelled heavy/light atoms and (iii) ligand validation.

### 3.1. Multimodal ADP distributions   

**Figure 1:**
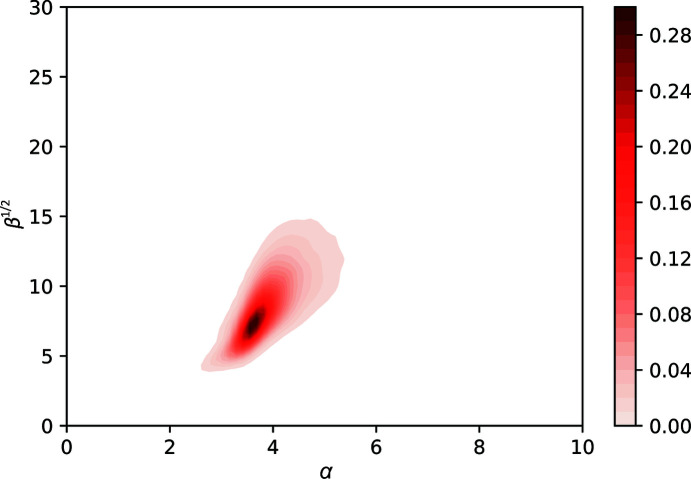
Smoothened α versus β^1/2^ plot for unimodal ADP distributions. Parameters are estimated using around 90 000 PDB entries after ten cycles of refinement. This plot was first presented by Masmaliyeva & Murshudov (2019 ▸) using around 45 000 PDB entries. The overall features of the plot are the same as those presented previously. This plot is used in *ToBvalid* for the validation of SIGD parameters.

The α/β plot reported previously (Masmaliyeva & Murshudov, 2019 ▸) was recalculated using 76 938 structures (Fig. 1 ▸) with unimodal ADP distributions; the overall features of the plot are the same as in the previous work.

For modes with large centroids, the β values and shift parameters (*B*_0_) are high. Also, the ADP distributions corresponding to these modes are more symmetric than those for modes with smaller centroids. There are at least two interrelated reasons for this: (i) as α and β become larger then, even without errors, the SIGD starts to resemble the Gaussian distribution and (ii) when ADPs are large they tend to have large errors. The ADPs correspond to the sum of two random variables: the ‘true’ ADP and errors in the estimation. As a result, under the naïve assumption that these two random variables are independent, the observed distribution becomes the convolution of an SIGD and a Gaussian distribution, again leading to a more symmetric distribution.

**Figure 2:**
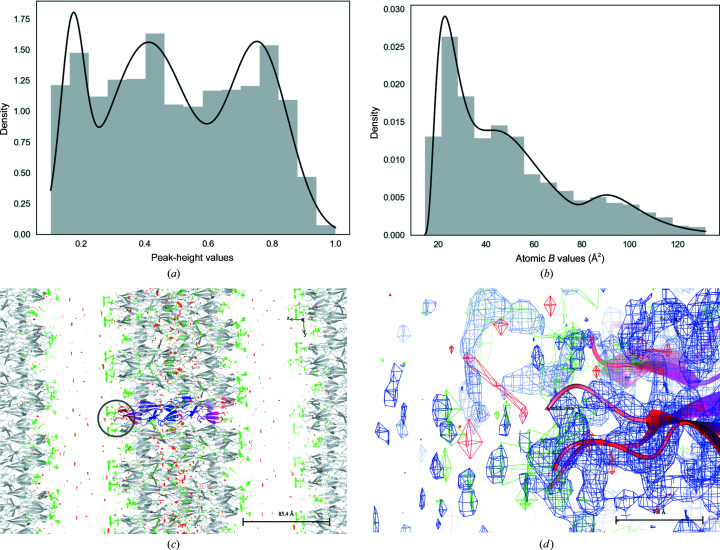
An example of a multimodal SIGD with three modes: PDB entry 5tu8 with disorder at both ends of the molecule causing multimodality in the ADP distribution. Presumably, in this case the whole crystal exhibits disorder. (*a*) Gaussian mixture model for peak-height distribution. (*b*) The mixture of SIGDs. (*c*) Continuous crystal for PDB entry 5tu8 showing disorder. The molecule in the asymmetric unit has been coloured for each cluster: from blue through magenta to red for low to high disorder. (*d*) An enlargement [marked by an oval in (*c*)] of the end of the molecule shows the presence of some positive density, although it would be a challenge to model it.

Estimation of multimodal ADP distributions shows that 13 902 out of 90 840 cases exhibit multimodality; most of them are bimodal. For the reasons given above, the second and higher modes are more symmetrical. There are only 266 PDB entries for which the ADP distributions show three modes. One such example is PDB entry 5tu8 (Fig. 2 ▸). Fig. 2 ▸ shows the Gaussian mixture model (GMM) for the PHD (Fig. 2 ▸ *a*) and the mixture of SIGDs (Fig. 2 ▸ *b*). In the case of PDB entry 5tu8, the crystal seems to be disordered. Part of the crystal does not have any interpretable density, presumably owing to the very high disorder of the molecules corresponding to this part. The first cluster of ADPs corresponds to the middle part of the molecule, whereas the second and third clusters correspond to the two opposite ends of the molecule where disorder starts. The parameters of the mixture of SIGDs for PDB entry 5tu8 are given in Table 3 ▸.

**Figure 3:**
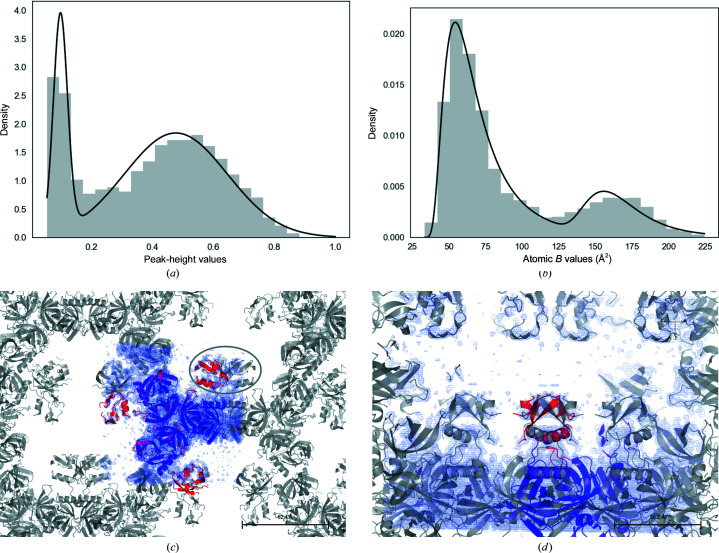
An example of a bimodal ADP distribution: PDB entry 4rqz. This protein has three domains. One of the domains makes contact with a single copy of its symmetry mate. This domain, together with its symmetry mate, has higher mobility than the rest of the molecule. (*a*) Gaussian mixture model for peak-height distribution. (*b*) The mixture of SIGDs. (*c*) Domains corresponding to the clusters in the ADP distribution. (*d*) Crystal contacts of the third domain of PDB entry 4rqz: an enlarged and rotated version of the region marked by an oval in (*c*).

In PDB entry 4rqz, there are two distinct modes (Figs. 3 ▸ *a* and 3 ▸ *b*). The molecule has three domains, two of which make contact with each other and their symmetry mates. These domains are responsible for crystal formation. The third domain only makes contacts with its symmetry copy (Figs. 3 ▸ *c* and 3 ▸ *d*). Since there are no other crystal contacts stabilizing them, this domain and its symmetry mate can move freely and therefore it has higher ADPs than the other domains.

Parameters of the mixture of *B*-value distributions for PDB entry 4rqz are given in Table 4 ▸. As expected, the density for the domains corresponding to the second mode is weaker than that for the first two modes (Fig. 3 ▸).

### 3.2. Local ADP analysis   

The algorithm described in Appendix *B* [App appb] was applied to all PDB entries considered. More than 1900 entries with a data resolution of 2 Å or better were manually analysed. More than 600 entries identified as potentially containing heavy atoms and their densities were carefully studied. The electron density corresponding to the atoms marked as light atoms is weaker and in many cases these atoms are exposed to solvent. As a result, in many cases the exposed atoms have higher ADPs than the surrounding atoms. Residues containing these atoms could have multiple conformations and might have been subjected to radiation damage. Analysis of radiation damage is outside the scope of this work.

**Figure 4:**
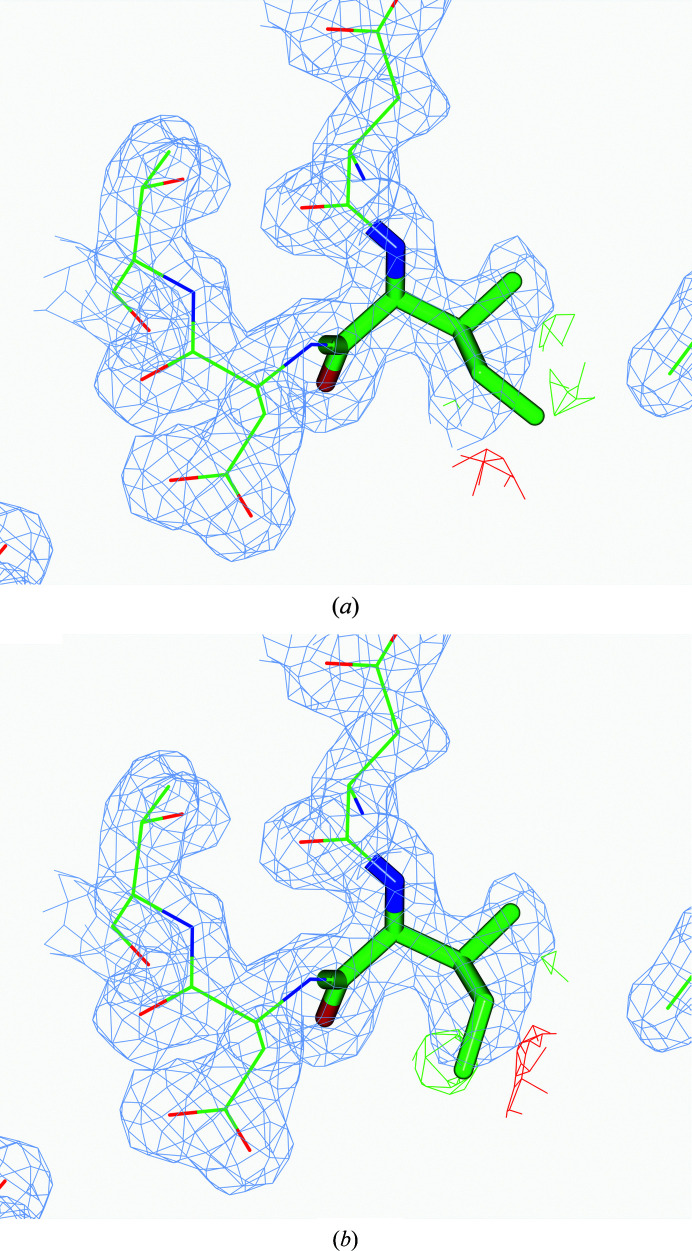
A potentially lighter atom than the surrounding atoms: the CD1 atom of Ile131A in PDB entry 2wxu. The incorrectly modelled rotamer was detected by the program as a lighter atom than the surrounding atoms. (*a*) The rotamer of Ile as present in the PDB file. (*b*) The rotamer of Ile after rebuilding.

Fig. 4 ▸ gives an example of an atom that is potentially lighter than the surrounding atoms (CD1 of Ile131A in PDB entry 2wxu). The calculated optimal occupancy is 0.64. The ADP of this atom is 37 Å^2^, whereas the median of the ADPs of the surrounding atoms is 20 Å^2^. Fig. 4 ▸(*b*) shows that this residue has been modelled in an incorrect rotamer. After rotamer correction using *Coot* (Emsley *et al.*, 2010 ▸) and subsequent refinement (Fig. 4 ▸ *b*), the ADP of the atom is 31 Å^2^ and the estimated occupancy has increased slightly to 0.7. There are still positive and negative densities around this residue, indicating that it might have multiple conformations. However, the existing data do not allow further accurate modelling of these.

**Figure 5:**
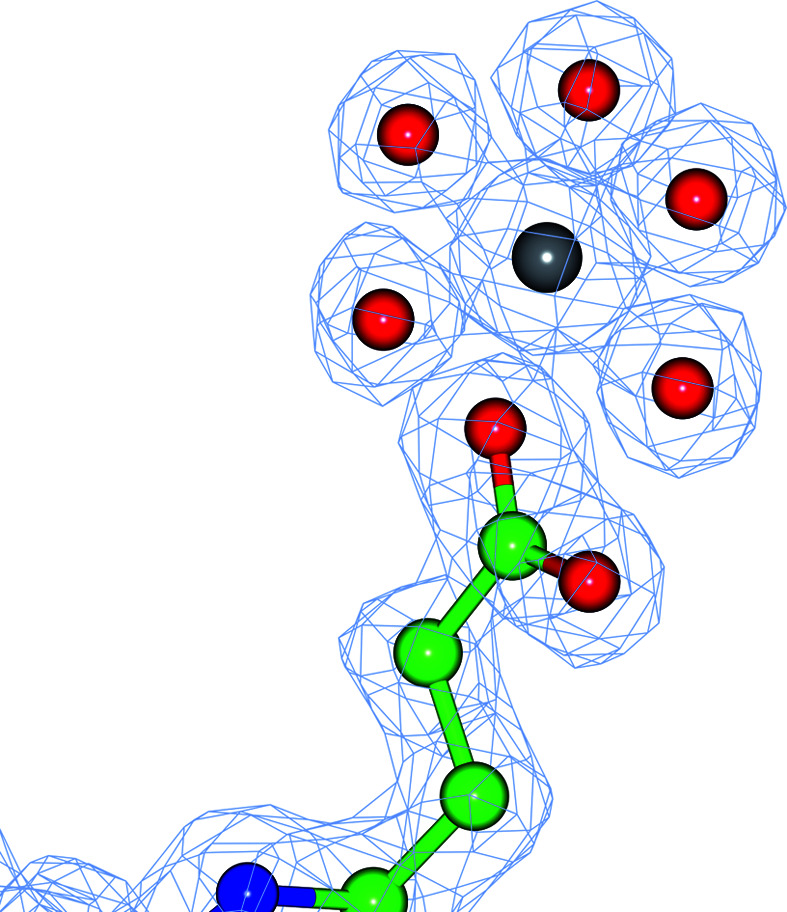
An example of a heavy atom modelled as a water molecule: residue 515F of PDB entry 2zbl. It is presumably an Mg^2+^ ion with six coordinating O atoms. The figure illustrates the Mg^2+^ ion in the position of the water after rebuilding and re-refinement.

Some metals are likely to be modelled as waters by automatic water-picking software, as such software does not usually analyse the interactions with the surrounding atoms and the height of the electron density when making decisions about the identity of atoms. The software usually looks for the existence of difference density. Several such cases have been identified in the examined PDB entries. Fig. 5 ▸ illustrates one such case. In the case of PDB entry 2zbl, water molecule 515F had six coordinating atoms forming an almost perfect octahedron. The ADP of this atom was 7 Å^2^ and the median ADP of the surrounding atoms was 15 Å^2^. This is one of the indicators that it may be a metal atom. The relative occupancy of this atom as calculated using (11) is 1.37. Two potential metal ions, Mg^2+^ and Na^+^, are considered further. Inspection of the crystallization condition showed that MgCl_2_ was used in the buffer. This would indicate that Mg^2+^ is more likely than Na^+^. Analysis of the distances between this atom and the surrounding atoms shows that they are between 2.09 and 2.2 Å. The ideal distance between Mg^2+^ and O is around 2.06 Å, and that between Na^+^ and O is around 2.35 Å. Taking these factors together suggests that this atom is Mg^2+^. Modelling it as Mg^2+^ followed by a few cycles of refinement yielded an ADP of 13 Å^2^ with an occupancy of 1.09 as estimated using (11).

**Figure 6:**
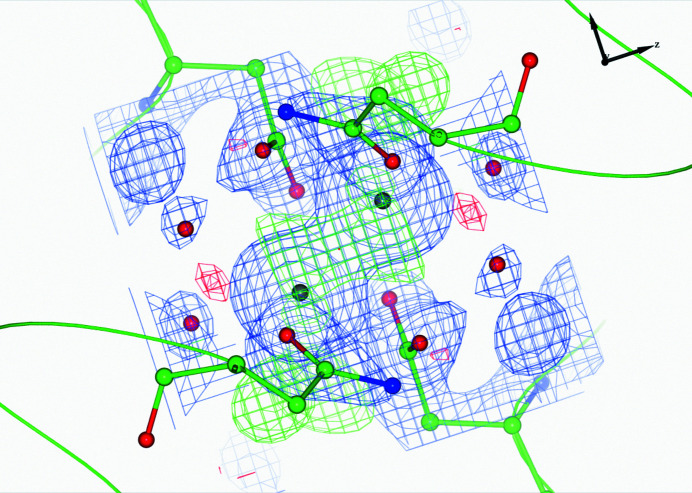
The Ca^2+^ atom (residue 1377) in chain *A* of PDB entry 2wxu was detected as being heavier than the neighbouring atoms. The figure illustrates the local neighbourhood of this ion after replacing the Ca^2+^ cation with a half-occupied Cd^2+^ cation at the same position. The twofold crystallographic symmetry axis is perpendicular to the plane and passes through the centre of the line connecting the heavy cations. The distance between symmetry-related Cd^2+^ ions is 2.3 Å, indicating that they cannot coexist. It is likely that when a Cd^2+^ is present in one position then the other position is occupied by a water molecule. As a result, the surrounding residues may also have multiple conformations. This also describes the positive density around the surrounding residues.

Many PDB entries contain heavy atoms, most of which seem to have the correct parameterization. An example of a PDB entry containing an incorrect parameterization is PDB entry 2wxu, in which residue 1377A is a Ca^2+^ cation with a relative occupancy of 1.36. The program marked this as a heavier atom with a *B* value of 14 Å^2^; the median *B* value of the neighbouring atoms is 25 Å^2^. The crystallization conditions contained CdSO_4_. Since this atom is close to a twofold-symmetry axis, its symmetry mate is at a distance of 2.3 Å and it was decided that the Cd^2+^ ion should have half occupancy. Refining this atom as Cd^2+^ with half occupancy gave an ADP of 18 Å^2^ for this atom, which is closer to those of its surroundings. After rebuilding using *Coot* (Emsley *et al.*, 2010 ▸) and re-refinement, this ion was no longer reported as an outlier. There were still some positive density around this position. This Cd^2+^ ion is close to its symmetry mate and the distance between them is 2.3 Å, which is close to the ‘ideal’ distance between Cd^2+^ and an O atom. This means that when Cd^2+^ is present at one of the positions the other position is occupied by a water molecule. The surrounding protein atoms also adjust to accommodate the Cd^2+^/water switch. The existence of multiple conformations also explains why the surrounding atoms have larger ADPs than the Cd^2+^ cation. Fig. 6 ▸ shows this atom, its symmetry mate and its coordination together with the map.

### 3.3. Application of local ADP analysis to ligand validation   

Local analysis was also applied to ligands. In this case all ligand atoms were considered and the median ADP of the ligands was compared with that of the neighbouring atoms. There were many cases in which ligands were marked as having substantially less than full occupancy. There were a number of SO_4_^2−^ and PO_4_^3−^ anions that did not seem to have supporting experimental evidence. These were not considered further. There were also a number of Zn and other metal atoms with suspicious density; as these have been considered by Touw *et al.* (2016 ▸) we did not analyse them further. More than ten PDB entries were inspected in detail, but only three of them were selected for this work. These are PDB entry 5x1o with the ligand I3P (inositol 1,4,5-trisphosphate), PDB entry 5orj with the ligand I6P (inositol 1,2,3,4,5,6-hexakiphosphate) and PDB entry 6b9b with the ligand MAL (maltose). Table 5 ▸ gives the relative estimated occupancies for these ligands together with the median ADPs of the ligands and the surrounding atoms.

#### 3.3.1. Case 1: PDB entry 5x1o   

**Figure 7:**
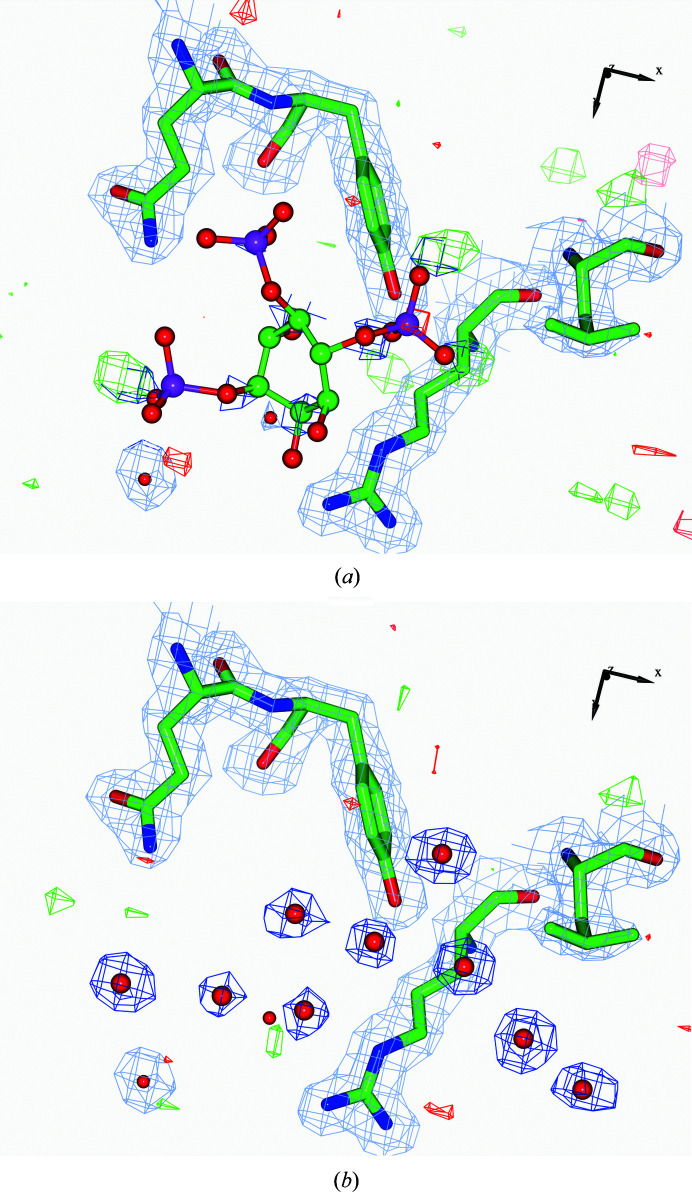
The I3P ligand of PDB entry 5x1o which was detected by *ToBvalid* as a potentially lighter ligand than the surrounding atoms. The high *B* values for the atoms of I3P and the resulting estimated low occupancy suggests that either this ligand is absent in the crystal or it is present with low occupancy. (*a*) The ligand before rebuilding after ten cycles of refinement. (*b*) After removal of the ligand and the placement of water molecules according to the difference map.

The estimated occupancy for the I3P ligand in this structure is 0.11, indicating that this ligand either is not present or is present with very low occupancy. Inspection of the electron density showed (Fig. 7 ▸ *a*) that there is no convincing electron density corresponding to this ligand. After removing it and adding water molecules where necessary the difference map became cleaner (Fig. 7 ▸ *b*).

#### 3.3.2. Case 2: PDB entry 5orj   

The estimated occupancy of the I6P ligand in this structure is 0.21, which again shows that it is either absent or present with low occupancy. Its median ADP is 252 Å^2^ and that of the surrounding atoms is 53 Å^2^. Inspection of the density and symmetry-related molecules showed that this ligand is on a twofold axis, resulting in non­bonding repulsions of symmetry-related molecules, moving them out of the density. After 40 cycles of refinement with half occupancy the median ADP of the ligand became 97 Å^2^, with that of the neighbours being 52 Å^2^, and its estimated occupancy became 0.6. The difference density became clean, although the density was still weak. This suggests that although the half-occupied ligand fits better there is still some disorder or mobility of this ligand. It is also a clear demonstration that crystal symmetries must be accounted for during model building and refinement.

For the map coefficients after refinement with fully and half-occupied I6P, see the supporting information.

#### 3.3.3. Case 3: PDB entry 6b9b   

**Figure 8:**
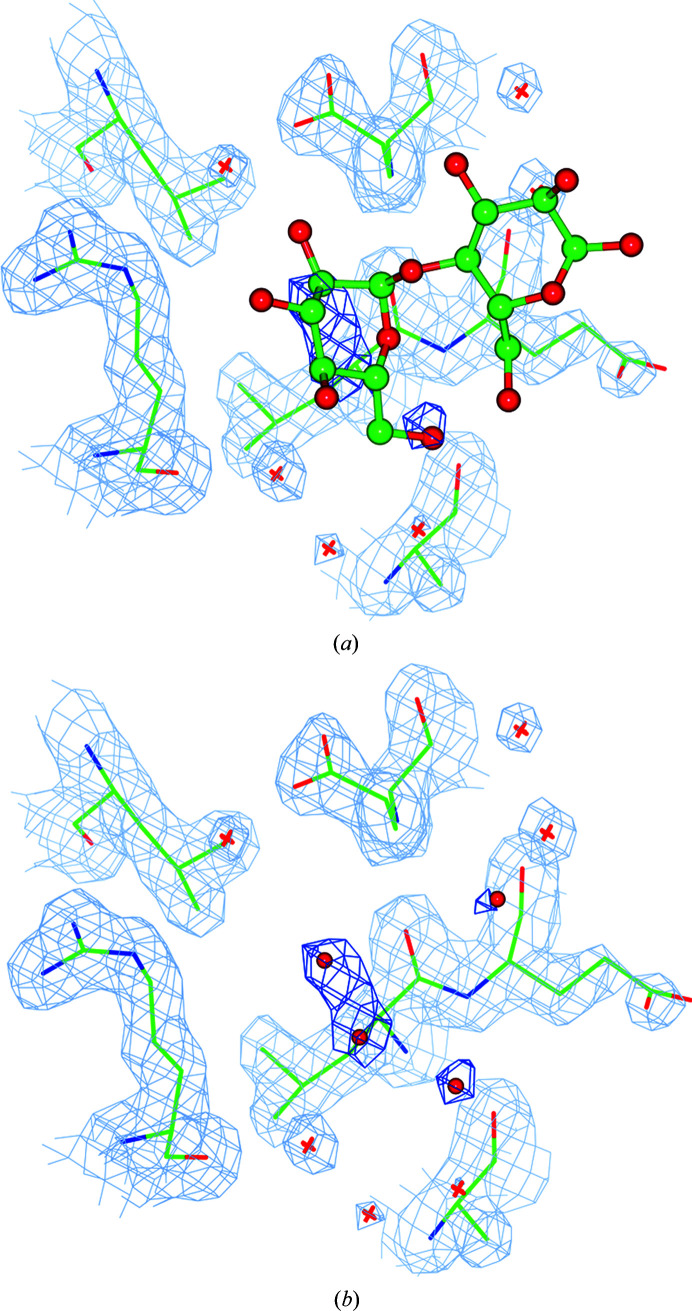
The MAL ligand of PDB entry 6b9b which was detected by the program as an outlier with low occupancy. There is no convincing density corresponding to MAL. The estimated low occupancy and the absence of convincing density suggests that this ligand does not exist in the crystal as a fully occupied molecule. (*a*) The ligand as present in the PDB after ten cycles of refinement. (*b*) The ligand was removed and water molecules were placed using the difference map.

The occupancy of the MAL ligand in the *B* chain of this structure is estimated to be 0.414. The electron density shows there is no convincing evidence that a fully occupied ligand is present in the crystal (Fig. 8 ▸ *a*). Refining the model without this ligand and adding water molecules according to the difference maps again cleaned up the density (Fig. 8 ▸ *b*).

All of these examples show that a comparative analysis of the ADPs of ligands with those of their neighbours can play a role in validation and has potential for the identification of incorrect or disordered ligands.

## 4. Conclusions   

Many macromolecular structures in the PDB solved by X-ray crystallography show multimodal distributions of ADPs. The ADPs of around 10% of the inspected PDB entries exhibited multimodality. The reasons for such behaviour are either incorrectly modelled parts of the structure or different domains having different intermolecular contacts. In both cases, the parts of the molecule corresponding to the modes with large average ADPs should be inspected. Such ADP distributions are modelled using a mixture of SIGDs. The Silverman method is used for identification of the number of modes and the expectation–maximization algorithm is used for parameter estimation. Multimodality may also indicate that the local resolvability in maps corresponding to different parts of the structure is different. In the limiting case, when an atom is placed in an incorrect position, the density and therefore the signal-to-noise ratio around that atom is very small. This results in very low local resolvability around the atom. Thus, analyses of the modes of the ADP distributions can shed some light onto the correctness, validity and mobility of different parts of the molecule, thus helping in the valid­ation and analysis of PDB structures. It may be expected that cryoEM structure models frequently exhibit multimodality, because the variation of local resolution in these structures has been well documented (Kucukelbir *et al.*, 2014 ▸).

The resolution- and ADP-dependent analysis of neighbouring atoms within structures has the potential to pinpoint mismodelled parts of the molecules. This can be used as a complementary validation tool during model building, refinement and deposition. Moreover, it can be used in the identification and modelling of metal ions. If used for the identification of metal atoms, the metal coordination should also be considered. The identified metal ions could be further checked using one of the metal-checking tools (Zheng *et al.*, 2017 ▸; Harding *et al.*, 2010 ▸) or by the direct use of bond-valence theory (Müller *et al.*, 2003 ▸; Brown, 2009 ▸; Harding *et al.*, 2010 ▸).

Comparative analysis of the ADPs of ligands and the surrounding atoms using the algorithm developed in this work allows the identification of potentially disordered and incorrectly modelled ligands. The approach described here uses the whole ligand as one unit. In practice, there are many cases in which only one part of the ligand is visible in the density. The algorithm can be extended to identify such cases by considering only local atom groups or local graphs describing parts of the ligands. It should be emphasized that the current algorithm does not provide information on whether the chemistry of a ligand is correct. Full and comprehensive ligand validation needs to consider the local chemistry, the stability of ligands, *B* values and density maps together. The program *ToBvalid* should be considered as a complementary tool to existing ligand-validation software packages (Tickle, 2012 ▸; Emsley, 2017 ▸).

The algorithms have been implemented in the program *ToBvalid*, which is available from https://github.com/ToBvalid/ as open-source software. The program can also be installed using the command pip install tobvalid.

All figures related to atomic models were generated using *CCP*4*MG* (McNicholas *et al.*, 2011 ▸).

## Supplementary Material

Comparison of `real atoms' and `point atoms'. DOI: 10.1107/S2059798320011043/ir5013sup1.pdf

Supplementary Data for PDB entry 5orj. DOI: 10.1107/S2059798320011043/ir5013sup2.zip

Description of the Supplementary Data. DOI: 10.1107/S2059798320011043/ir5013sup3.pdf

## Figures and Tables

**Table 1:** PDB entries rejected from analysis Note that multimodal cases are considered further for modelling using a mixture of SIGDs.

No. of remaining entries	No. rejected	Reason for rejection
127708	12935	Re-refinement failure†
114773	19509	Outside 1.5–3 Å resolution
95264	1491	High *R* factor
93773	2138	Viruses
91635	795	No. of atoms *n* < 500
90840	13902	Multimodal
76938	—	—

† Restrained refinement was applied.

**Table 2:** Summary of the PDB entries used as examples *R* and *R*_free_ before, *R* factors before refinement; *R* and *R*_free_ after, *R* factors after refinement.

PDB code	Case	Resolution (Å)	*R* before	*R* after	*R*_free_ before	*R*_free_ after	α	β	*B*_0_ (Å^2^)
5tu8	Three modes	2.33	0.210	0.201	0.240	0.250	3.49	120.58	2.31
4rqz †	Bimodal	2.40	0.196	0.202	0.226	0.239	2.63	110.94	26.00
2wxu	Lighter atom, heavier atom	1.80	0.175	0.175	0.216	0.216	4.69	95.37	3.58
2zbl	Heavier atom	1.60	0.152	0.135	0.182	0.160	4.85	51.89	1.32
5x1o	Wrong ligand	1.90	0.225	0.252	0.276	0.300	3.46	60.90	3.59
5orj	Wrong ligand	1.99	0.200	0.209	0.221	0.244	3.96	149.25	14.01
6b9b	Wrong ligand	1.80	0.135	0.140	0.162	0.166	3.69	42.43	10.37

† SIGD parameters for multimodal cases are given for unimodal parametrization in this table.

**Table 3:** Parameters of the SIGD mixture for PDB entry 5tu8

Distribution	1st	2nd	3rd
Mix parameters	0.52	0.37	0.11
α	3.55	10.06	7.5
β	53.59	481.24	373.23
Shift	10.82	6.13	59.56
Mean	30.73	58.77	100.67

**Table 4:** Parameters of the SIGD mixture for PDB entry 4rqz

Distribution	1st	2nd
Mix parameters	0.79	0.21
α	3.74	10.65
β	122.74	726.67
Shift	28.26	99.82
Mean	72.34	174.3

**Table 5:** Ligand-validation results

PDB code	5x1o	5orj	6b9b
Resolution (Å)	1.9	1.99	1.8
Ligand, residue No., chain	I3P, 201, *A*	I6P, 407, *A*	MAL, 807, *B*
Optimal occupancy (total density)	0.12	0.21	0.41
Optimal occupancy (peak height)	0.09	0.11	0.27
Median *B* of the ligand (Å^2^)	125	252	99
Median *B* of the environment (Å^2^)	12	53	37

## Appendix A Estimation of the parameters of multimodal *B*-value distributions

Let **B** be a vector of the sample of the data that comes from the population with the probability distribution as a mixture,

where π_*j*_ are the mixture parameters and θ_*j*_ are the parameters of φ(*B*, θ) corresponding to the mode *j*, φ(*B*, θ) is the parametrized family of distributions, *N* is the number of data points and *N*_mode_ is the number of modes. In the case of ADPs, the parameterized distribution is the SIGD (2). Estimating the parameters of (13) directly is numerically unstable as extremely small and large values are summed together. To circumvent this problem, an additional vector of a random variable **Z** that is the extent to which each point belongs to different modes is introduced. The resulting probability distribution of the augmented model is then

As a result, we have a product of the density of distribution which is easier to optimize; however, new unknown parameters **Z** have been introduced. This problem can now be solved using the expectation–maximization algorithm (Dempster *et al.*, 1977 ▸; Bishop, 2006 ▸): estimate **Z** as the expectation of the posterior probability distribution *P*(**Z**|**B**, **θ**) and estimate the parameters **θ** by maximum-likelihood estimation using the distribution *P*(*B*|**Z**, **θ**) with fixed **Z**. The algorithm for solution of this problem is well known (see, for example, Bishop, 2006 ▸). Here, we adapt this algorithm to estimate the parameters of the mixed SIGD.

(1) Estimate the number and the centroids of the clusters using Silverman’s test for multimodality (Silverman, 1981 ▸) as implemented in *SciPy*.
(2) If *N*_mode_ > 1 then calculate the peak heights using (3). This gives the peak-height distribution. Applying the expectation–maximization algorithm with the Gaussian mixture model (Bishop, 2006 ▸), estimate the posterior distribution of the extent to which each atom belongs to each mode: *z_ij_*, *i* = 1…*N*, *j* = 1…*N*_mode_.
(3) Using (*z_ij_*) apply the EM algorithm to the SIGD mixture model.
  (*a*) Estimate the initial parameters of SIGD.
    (i) Mixture parameters:
    (ii) Find the mean and minimum of *B* for each group:
      Here *b* is a very small positive number.
    (iii) Set parameters:
  (*b*) Expectation step:
  (*c*) Maximization step. In this step we use only *z_ij_* for which *B*_*i*_ > *B*_0,*j*_; otherwise, the corresponding *z_ij_* are set to 0. To avoid negative arguments in the logarithms this fact is accounted for during summation. For each mode, calculate the derivatives and perform maximization. Note that the formulas are the same as those in Masmaliyeva & Murshudov (2019 ▸) except that they are now applied for each mode.
    (i) The negative log-likelihood function has the form
    (ii) The first derivatives have the form
    (iii) The expected Fisher information matrix has the form
    (iv) Find the shifts *s* = −*I* ^−1^ *g*, where
      and apply them to the parameters (α_*j*_, β_*j*_, β_0,*j*_).
    (v) Repeat the calculations until convergence.
  (*d*) Repeat (*b*) and (*c*) until convergence.

The EM algorithm is well known and converges well if the number of modes is known and the parameters are close to the maximum-likelihood solutions. We use pre-processing of the data using the peak-height distribution (PHD), the Silverman method for mode identification and initial Gaussian mixture model estimation for the PHD. To reduce the dependence on the initial parameter estimation, we use the stochastic EM algorithm (Diebolt & Celeux, 1993 ▸), which is known to have better convergence properties than the classical EM. A user can select the classical or stochastic EM algorithm. Here, we give the values corresponding to the latter. The program was applied to more than 90 000 PDB entries used in this work. In all cases, when the number and approximate positions of the modes are identified accurately, the algorithm converged in a reasonable time. For example, on a computer with an i7 Intel core 2.3 GHz processor it took 29 iterations and 1.3 s to converge for PDB entry 6et7 with 10 500 atoms, for which there were two modes. For PDB entry 2pan with 28 000 atoms and two modes of the distribution, it took 81 iterations and 9.1 s.

## Appendix B Local *B*-value analysis

The estimation of the occupancy of an atom in relation to its surroundings is performed using the total density difference (11) and simple statistics. The procedure consists of three steps.

(i) The number and list of neighbours of each atom are calculated using the *GEMMI* library (Wojdyr, 2017 ▸); an interatomic distance equal to 4.2 Å is used as a default parameter. This can be adjusted by the user as an input parameter to the program *ToBvalid*.
(ii) If an atom has three or more neighbours, it is tested further. Values of the relative peak height at the centre of atoms are calculated using (11) for the atom in relation to its neighbours. Let the corresponding relative occupancies of the atom be *c*_0_, *c*_1_ and *c*_3_ with respect to the median, first and third quartiles of ADPs of the neighbouring atoms. If *c*_0_ > 1.2 and *c*_1_ > 1.01 then the atom is considered to be heavier than its neighbours; if *c*_0_ < 0.8 and *c*_3_ < 0.99 then this atom is considered to be lighter than its neighbours. In both cases the optimal occupancy *c*_0_ is reported. These parameters are default values that have been selected by trial and error.
(iii) If the inspected atom is an O atom of a water molecule and it has six or more neighbours then it is marked as an atom with unusual behaviour. These are the candidates that are considered as metals.
