# Supplementary material for: Local and global analysis of macromolecular atomic displacement parameters
Source: Acta Crystallogr D Struct Biol. 2020 Sep 22;76(Pt 10):926–37. doi: 10.1107/S2059798320011043 (PMC7543658; doi:10.1107/S2059798320011043)
Supplement: Supplementary file 1 [file d-76-00926-sup1.pdf]

STRUCTURAL BIOLOGY

**Volume 76 (2020)**

**Supporting information for article:**

# Local and global analysis of macromolecular atomic displacement parameters

**Rafiga C. Masmaliyeva, Kave H. Babai and Garib N. Murshudov**

## S1. Local B value differences and atomic scattering factors

### S1.1. General formulas

In the main text we used local ADPs and point atoms to identify locally interesting/mismodelled parts in atomic models. Here we give formulas that account for atomic identities. Since the scattering factors are dependent on the experimental method we describe this for general case and potential practical implementation details at the end of this subsection. We will consider only formulas (10) related to the total density differences. Formulas for comparative analysis of peak heights (5) can be derived similarly.

The formula we are interested in is:

$$\int_{|s|<s_{\max}} \left(f_1(s) - c f_2(s)\right)^2 ds \rightarrow \min \quad \text{(S1)}$$

Which is minimised when:

$$c = \frac{\int_{|s|<s_{max}} f_1(s) f_2(s) ds}{\int_{|s|<s_{max}} f_2^2(s) ds} \qquad \text{(S2)}$$

Using spherical polar coordinates and assuming that scattering factors do not depend on directions then we can write:

$$c = \frac{\int_0^{s_{max}} s^2 f_1(|s|) f_2(|s|) d|s|}{\int_0^{s_{max}} s^2 f_2^2(|s|) d|s|} \qquad \text{(S3)}$$

For X-ray crystallography we will use the sum of Gaussians approximation for form factors of atoms without B values.:

$$f_0(|s|) = \sum_{i=1}^{N_{gauss}} a_i e^{-\frac{b_i |s|^2}{4}} \qquad \text{(S4)}$$

Then for atom with a B value equal to B will have a scattering factor

$$f(|s|) = \sum_{i=1}^{N_{gauss}} a_i e^{-\frac{b_i |s|^2}{4}} \, e^{-\frac{B|s|^2}{4}} \qquad \text{(S5)}$$

Putting (S3) in (S1) results in:

$$c_{X-ray} = \frac{\int_0^{S_{max}} |s|^2 (\sum_{i=1,j=1}^{N_{gauss},N_{gauss}} a_{1,i} a_{2,j} e^{-(b_{1,i}+b_{2,j}+B_1+B_2)|\frac{s^2}{4}} d|s|}{\int_0^{S_{max}} |s|^2 \left(\sum_{i=1,j=1}^{N_{gauss},N_{gauss}} a_{2,i} a_{2,j} e^{-\left(b_{2,i}+b_{2,j}+2B_2\right)\frac{|s|^2}{4}}\right) d|s|} \qquad \text{(S6)}$$

This formula can further be simplified and brought in to the form of (10). The expression is given in the next subsection.

Now if we know the identity of the second atom then for the first atom we would need to scan over all potential atoms, calculate corresponding *c* and then put the results in (S1). If this technique is used then we would select the atom that results in the minimum value of (S1).

Since scattering factors for neutron diffractions do not depend on resolution the resultant calculations would be slight modification of

$$c_{neutron} = \frac{f_1}{f_2}\left(\frac{2B_2}{B_1+B_2}\right)^{\frac{3}{2}} \frac{-s_{max}\sqrt{B_1+B_2}e^{-\frac{(B_1+B_2)s_{max}^2}{4}}+\sqrt{\pi}\,\mathrm{erf}\left(\frac{\sqrt{B_1+B_2}s_{max}}{2}\right)}{-s_{max}\sqrt{2B_2}e^{-\frac{2B_2s_{max}^2}{2}}+\sqrt{\pi}\mathrm{erf}\left(\frac{\sqrt{2B_2}s_{max}}{2}\right)} \quad \text{(S7)}$$

Where $f_1$ and $f_2$ are form factors for elements corresponding to atom 1 and 2.

For electron diffraction we can use the Mott-Bethe formula

$$f_e(s) = \frac{Z-f_X(s)}{|s|^2} \quad \text{(S8)}$$

Where $f_e$ is the scattering factor for electrons and $f_X$ is the scattering factors for X-ray diffraction. In this case the formula for "estimated occupancies" become (we do not the expression further

$$c_{electron} = \frac{\int_0^{s_{max}} \frac{(Z_1-f_{1,X}(s))(Z_2-f_{2,X}(s))}{|s|^2} e^{-\frac{(B_1+B_2)|s|^2}{4}} d|s|}{\int_0^{s_{max}} \frac{(Z_2-f_{2,X}(s))^2}{|s|^2} e^{-\frac{B_2|s|^2}{2}} d|s|} \quad \text{(S9)}$$

Integrals can be evaluated by numerical integration. If this formula is implemented then due attention must be paid to the fact that if $B_1$=$B_2$ = 0 then integrals related to the charged atoms may diverge and the effect of divergence are cancelled only if charges of involved atoms are the same. In general, accounting for charges in electron diffraction is a tricky problem as charge redistribution as well as charge screening effects should be accounted for.

One also should account for the fact that these integrals are best evaluated as they are without expanding into components. If involved sums are expanded and evaluated individually then all involved integrals will diverge.

### S1.2. Application to ADPs of the atomic models derived using X-ray diffraction

In case of the X-ray crystallography $N_{gauss}$ Gaussians for the form factors have the form (S4). If we assume that the current modelled atom has the form factor $f_1$(s) and ADP equal to $B_1$ and we think that the atom should be with the form factor $f_2$(s) and ADP equal to $B_2$ (surrounding atoms) then to estimate the "occupancy" we first normalise the form factor. I.e. we replace the coefficients with $a_i/\sum_{j=1}^{N_{gauss}} a_j$. This is done to ensure that the results for different atoms can be compared. If it is decided that the atom must be one with the form factor $f_2(s)$ then "actual" occupancy should be calculated using the formula:

$$c_{actual} = c_{X-ray} \frac{\sum_{i=1}^{N_{gauss}} a_{1i}}{\sum_{i=1}^{N_{gauss}} a_{2i}} \quad \text{(S10)}$$

the formula for the "estimated occupancy" has the form (it is just a different form of (S6)):

$$c_{X-ray} = \frac{\sum_{i=1,j=1}^{N_{gauss}} a_{1i}a_{2j}ph(b_{1i}+b_{2j}+B_1+B_2,s_{max})}{\sum_{i=1,j=1}^{N_{gauss}} a_{2i}a_{2j}ph(b_{2i}+b_{2j}+2B_2,s_{max})} \quad \text{(S10)}$$

Where $a_{ij}$ and $b_{ij}$ are the parameters of the Gaussians of the form factors, and *ph(B,s)* has the form (it is the same as formula (10) in the main text):

$$ph(B,s) = \left(\frac{4\pi}{B}\right)^{\frac{3}{2}}\left(-s\sqrt{B}e^{-Bs^2/4} + \sqrt{\pi}\,erf(\sqrt{B}s/2)\right)$$

Figure S1 shows the “estimated occupancies” for different atom types. As it can be expected the estimated occupancies depend on the resolution, ADP differences as well as atom identities. Although point atom approximation somewhat overestimates the occupancies, general tendencies of the estimated occupancies are the same for all cases.

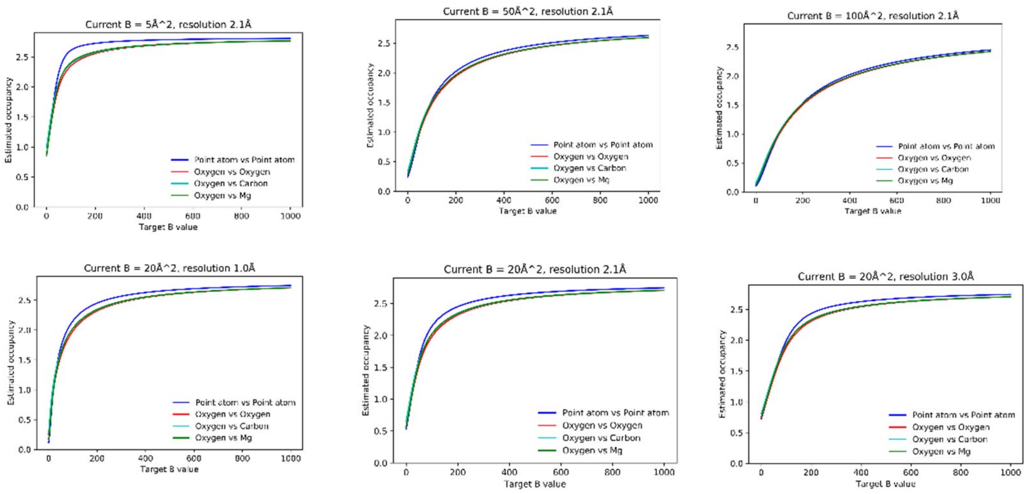


**Figure S1** Occupancy estimation using “real atom” X-ray scattering form factors for different ADPs and resolutions. It is seen that a point atom approximation gives a general tendency of the “estimated occupancies”. Although, they somewhat overestimate the occupancies when the atom under consideration has lower B value than surrounding ones. Note that different “real atoms” give similar estimation. It is likely that the reason is that “real atoms” do have width even at infinity resolution and ADP equal to 0. As it could be expected, these plots also show that there is a dependence of the estimated occupancies on resolution as well ADPs.
