# Supplementary material for: Local and global analysis of macromolecular atomic displacement parameters
Source: Acta Crystallogr D Struct Biol. 2020 Sep 22;76(Pt 10):926–37. doi: 10.1107/S2059798320011043 (PMC7543658; doi:10.1107/S2059798320011043)
Supplement: Supplementary file 3 [file d-76-00926-sup3.pdf]

STRUCTURAL
BIOLOGY

Volume 76 (2020)

Supporting information for article:

Local and global analysis of macromolecular atomic displacement parameters

Rafiga C. Masmaliyeva, Kave H. Babai and Garib N. Murshudov

The list of files for the pdb entry 5ORJ

There are four files:

1) 5orj_before.pdb and 5orj_before.mtz correspond to the refinement outputs with fully occupied ligand - I6P.
2) 5orj_after.pdb and 5orj_after.mtz correspond to the refinement results with half occupied ligand – I6P.
